# Observations Based upon Five Hundred Operations for Appendicitis1Read before the Indiana State Medical Association, May 24, 1906.

**Published:** 1906-10

**Authors:** Thomas B. Eastman

**Affiliations:** Indianapolis, Ind.


### SELECTION.

### Observations Based upon Five Hundred Operations for Appendicitis.[1]

BY THOMAS B. EASTMAN, M. D., Indianapolis, Ind.

[*From The Lancet-Clinic.*]

IT is the purpose of this paper to note certain observations made by the writer in a series of five hundred cases of appendicitis, the operations having been performed either by the writer or his

1. Read before the Indiana State Medical Association, May 24, 1906.

brother, Dr. Joseph Rilus Eastman. For the most part, the ideas expressed are in accord with the accepted teaching; but where they do not accord with such teaching, no apology is offered, as the paper purports to represent nothing more or less than opinions of such value as may be formed in a careful study of such a number of cases. No radical views are expressed, and while some competent operators may disagree with all or part of the writer's ideas, they at least have whatever value may attach to an operating room rather than to a laboratory or deadhouse experience.

The importance of prompt and early diagnosis can hardly be overestimated, and that the mortality following operations for appendicitis has been so greatly reduced during the past few years is due in no small degree to the attending physician, who, from his own clinical observations and from the teaching of operators, is gradually coming to realize that there is a time above all others when operation should be made.

The refusal of the patient or family to submit to operation has often served to delay recommended measures for relief, but the attending physician has not always appreciated his responsibility in this matter, and it is a source of regret that some have not yet learned the lesson. On the other hand, the number of those physicians who have seen fit to place themselves behind the shield of what they are pleased to call "conservatism" is most happily decreasing. We take it that to be conservative means to conserve life, and, so far as this disease is concerned, we are fully satisfied that this false conservatism has cost vastly more lives than has the most rampant radicalism.

The division of the course of the disease into hours and days according to its duration is not always reliable, as it is readily conceived that pathological processes do not proceed by the clock, but for purposes of convenience we may consider the time for operative procedure in four periods: First, within the first forty-eight hours; second, from the end of this period to the fifth or sixth day; third, from the sixth day on; and fourth, the interval. At the present day, one may say without fear of any considerable opposition that the early operation is unquestionably and unequivocably the operation of election. The condition in and around the appendix, the limited area of inflammation, the absence of adhesions, the absence of pus, the fact that we operate almost surely before perforation or gangrene shall have occurred, the general condition of the patient, the unlowered vitality, the uninvaded lymphatics, and last, but not least, the low mortality resulting from operations made in this stage by competent men, seem to the writer almost to preclude argument upon this question.

It is in the second stage, however, where it behooves the surgeon to use his most careful judgment as to the time for surgical intervention. In this stage, while the operation should be made in a large majority of cases, the dictum, "operate as soon as the diagnosis is made," cannot be absolute.

Take a case of appendicitis of seventy-two hours duration, which is not to be operated, and consider what the subsequent history of the case may be. The further pathologic processes may be divided into two classes : first, those favorable to the patient ; and second, those unfavorable. Among those favorable, we have:

1. The infectious material may escape into the cecum via the appendix.

2. The inflammation may become circumscribed and limited to the lymphatics in the immediate vicinity of the appendix.

3. There may result an extra-appendiceal abscess which may rupture into an adherent coil of intestine.

4. There may result a quiescent abscess.

Among those unfavorable:

1. There may develop a diffuse general suppurative peritonitis.

2. If there be a circumscribed abscess, there may be an iliac thrombo-phlebitis and infection of the venous circulation, resulting in embolism infarcts and secondary abscess.

3. A portal venous branch may thrombose and hepatic abscess or pylephlebitis result.

4. The infection may by various channels reach the genito-urinary tract, gallbladder, liver, or lung (after Murphy).

In so far as those conditions which have been noted above as unfavorable are concerned, it is readily conceded that the sooner operation is made the bettter. The same may be said concerning certain of those conditions in the class noted as favorable, but the cases which should be considered as exceptions are those in which the patient shows but little general invasion of the infection, the pulse has continued good, the temperature curve is satisfactory, the secretions or excretions active and the patient's general condition good. In these cases, while it may be realized that an abscess is in the process of development, it is at the same time evident that nature is more than holding his own against the disease.

Organic adhesions do not develop under five days, and in a certain proportion of cases, where nature is thus walling off a pus cavity and protecting the abdominal cavity, it is occasionally unwise to interfere and break up these adhesions. The wisdom of the old maxim, "*ubi pus ibi evacua,*" is not questioned, but the wisdom of evacuating this pus, as must often be done by the best operators, into the free and hitherto uncontaminated abdominal cavity, is most seriously questioned. In many of these cases, let alone, the abscess sac becomes adherent to the abdominal wall and the best of drainage may be accomplished by a simple incision through this structure, or, at least, it develops in such a way as to render drainage possible without entering the cavity. In the third and fourth classes of cases there is generally no question as to the advisability of operation, but even some of these third-class cases may be left to the interval.

In cases where we do not expect to find pus or use drainage, we employ the gridiron incision, severing all structures in the direction of their fibres, and in this connection it is well to point out that in enlarging such an incision the tendency to cut transversely the fibres of the muscles, the operator not taking the trouble to split the muscle fibres, as was done in the initial operation, is bad, as such procedure is likely to result in an unnecessarily weakened abdominal wall. In abscess cases the incision is made straight through the wall, and its location with reference to the abscess sac depends upon whether or not the sac is expected to be found adherent to the abdominal wall or whether we expect to evacuate the abscess well toward the flank, as it were, between the weather boards and the plaster.

The gridiron incision is impracticable in abscess cases, since being, in a way, a self-closing incision, it interferes with the drainage. We make large incisions to the end that we may have a good view of all the tissues involved in the pathologic process. The incision made, the appendix is located as follows: Placing the finger upon the peritoneum at the right of the incision and following the peritoneum around the flank, the first intestine to come in contact with this finger must necessarily be the lower portion of the ascending colon. The cecum is then easily grasped, withdrawn through the wound, and the appendix exposed, or if the cecum and appendix be adherent, the latter may easily be located once the cecum is thus under the finger. In our opinion, it is important to withdraw the cecum sufficiently well up to have the appendix well in view. To attempt to work in the abdominal cavity not only tends to render the procedure more difficult, but renders infection more probable. But little cecum need be exposed, and that little is easily protected. Of course, this cannot always be done where adhesions are dense. In clamping the appendix, we use either a simple hemostatic clamp or the forceps with the protecting shield designed by my brother. In cutting off the appendix with the cautery, the heat imparted to the jaws of the forceps tends to sterilize all the stump within the grasp of the forceps. Where we use a simple clamp, we do not use carbolic acid to cauterize the stump, because, on account of the small amount of tissue reached by the acid, this procedure looks like child's play.

In tying off the meso-appendix it is well not to have the ligature approach the wall of the appendix too closely, for, if it does, it renders the turning in of the stump difficult. If it is introduced close to the wall of the appendix, the meso-appendix should be dissected down to a point at least a quarter of an inch below where the lower border of the clamp is to be placed.

In our work on the appendix, as well as in all our intestinal work, for the twelve months preceding February 1, we had used the Pagenstecher celloiden linen. Since the latter date we have used von Brun's hemp. Either one of these suture materials is is vastly superior to catgut for several reasons. They are much more easily manipulated, they do not curl up in the hands, they tie with a firmer and smaller knot, their caliber is much smaller, they allow of a much more accurate coaptation of the parts, they are of such firm texture as to render invasion of bacteria improbable, they are of positive tensile strength, and are capable of unquestionable sterilisation.

It is important that the purse-string suture be placed at sufficient distance from the clamp to insure perfect inversion of the appendix. The cecum is generally large enough, so there need be no fear of narrowing the lumen. We regard the placing of a ligature around the stump as bad practice, as it leaves an infected area tied off and with no chance of drainage. We have in certain cases, owing to the dense adhesions which rendered the withdrawal of the cecum from the cavity impossible, in emergency operations by lamp-light and in diffuse septic peritonitis, trusted to the simple ligature of the appendix. However, in all these cases we have used drainage. We leave the purse-string suture and the ligature around the meso-appendix long and bring them together in such a way that the cut edge of the meso-appendix lies in contact with the cecum at the point from which the appendix has been removed, and thus leave absolutely no raw surfaces within the cavity.

The technic in simple not infected cases has reached a stage where it may be considered satisfactory. It is in the infected cases where we may hope for improvement. These cases may be divided, according to Van Buren Knott, into three classes: First, the more or less localised peritoneal infection in which the general peritoneal cavity is not protected by limited adhesions; second, an infection sharply localised and circumscribed, the general cavity of the peritoneum being protected by a wall of adhesions; third, a diffuse, widespread infection of the entire peritoneal cavity.

The first class embraces those cases in which, as stated above, we occasionally see fit to delay. Oftentimes the existence of this condition is not suspected. The appendix is removed, and every point to which fluids may gravitate is carefully inspected and as carefully mopped out. In females a vaginal puncture is made and drainage instituted by this route. In males we use a large split rubber drainage-tube carrying gauze, making sure that it not only reaches the dependent pouches, but that it will remain there, and that, if possible, the movements of the intestines will not bend it so as to obstruct the drainage. The tube should be of stiff and not easily collapsible rubber. We never trust to simple gauze drainage, for the reason that it rarely drains. Oftentimes hernia will result from the use of large drainage-tubes, but what doth it profit a man if he prevents a hernia and gets a funeral.

In the second class—that is, where the infection is well walled off—our one aim is to drain the abscess without exposing the general abdominal cavity, and with careful study as to the point for incision this can be more commonly done than is generally believed. Where the drainage cannot be instituted without invasion of the general cavity, we feel that considerable time spent in building up a cofferdam is time well spent. Unless the appendix presents plainly, and can be removed easily, we do not remove it, and make a habit of stating to the patient prior to the operation that he or she may require a second operation for the cure of the hernia or removal of the appendix, or for both. In cases where the general cavity has been avoided, simple drainage by tube covered gauze is used, but where we have invaded the peritoneal cavity we take pains to see that the abscess drains externally and not into the cavity.

In the third class, that of diffuse septic peritonitis, wherein we have that vicious form of inflammation which involves the whole peritoneum, there are two cardinal points in procedure—rapidity of operating and drainage that drains. We make a free median incision, and, if the patient has strength enough to warrant it, we remove the appendix, simply tying off the meso-appendix and the appendix without any attempt at inversion. We then cleanse the cavity, looking well into all dependent pouches. In the female we use the vaginal puncture and drain as above described. In the male we place the tube covered gauze in the recto-vesical pouch and out the lower angle of the incision. In short, we do as little as we possibly can and still remove the cause and establish thorough drainage. In all our abscess cases we place our patient in the exaggerated Fowler position, and believe that the introduction of this detail in the after-treatment of appendicitis has had much to do with the present lowered mortality.

